# The Anaphase Promoting Complex Contributes to the Degradation of the *S. cerevisiae* Telomerase Recruitment Subunit Est1p

**DOI:** 10.1371/journal.pone.0055055

**Published:** 2013-01-25

**Authors:** Jenifer L. Ferguson, William Chong Hang Chao, Ethan Lee, Katherine L. Friedman

**Affiliations:** 1 Department of Biological Sciences, Vanderbilt University, Nashville, Tennessee, United States of America; 2 Section of Structural Biology, Institute of Cancer Research, London, United Kingdom; 3 Department of Cell and Developmental Biology, Vanderbilt University, Nashville, Tennessee, United States of America; Tulane University Health Sciences Center, United States of America

## Abstract

Telomerase is a multi-subunit enzyme that reverse transcribes telomere repeats onto the ends of linear eukaryotic chromosomes and is therefore critical for genome stability. *S. cerevisiae* telomerase activity is cell-cycle regulated; telomeres are not elongated during G1 phase. Previous work has shown that Est1 protein levels are low during G1 phase, preventing telomerase complex assembly. However, the pathway targeting Est1p for degradation remained uncharacterized. Here, we show that Est1p stability through the cell cycle mirrors that of Clb2p, a known target of the Anaphase Promoting Complex (APC). Indeed, Est1p is stabilized by mutations in both essential and non-essential components of the APC. Mutations of putative Destruction boxes (D-boxes), regions shown to be important for recognition of known APC substrates, stabilize Est1p, suggesting that Est1p is likely to be targeted for degradation directly by the APC. However, we do not detect degradation or ubiquitination of recombinant Est1p by the APC *in vitro*, suggesting either that the recombinant protein lacks necessary post-translational modification and/or conformation, or that the APC affects Est1p degradation by an indirect mechanism. Together, these studies shed light on the regulation of yeast telomerase assembly and demonstrate a new connection between telomere maintenance and cell cycle regulation pathways.

## Introduction

Telomeres are unique protein-DNA complexes found at the termini of linear eukaryotic chromosomes. These regions are critical for protecting chromosomes against nucleolytic digestion and for distinguishing normal chromosome ends from internal double-strand breaks. Loss of telomere function causes end-to-end fusions that result in anaphase bridge-breakage cycles and catastrophic genomic instability [1]. While the majority of the telomere is comprised of tandem G/T-rich double-stranded DNA repeats, the terminus exists as a short 3′-overhang throughout the cell cycle [2], [3]. After passage of the replication fork in late S phase, the 3′-overhangs are transiently increased in length, at least in part due to exonucleolytic digestion of the 5′-strand [2]–[5]. Telomerase, a ribonucleoprotein complex, can extend these 3′-overhangs by reverse transcription, while the conventional lagging-strand DNA replication machinery is thought to fill in the 5′-gap [6].

*S. cerevisiae* telomerase contains three dedicated protein subunits (Est1, Est2 and Est3) [7]–[9] and an intrinsic RNA (*TLC1*) containing the template for nucleotide addition [10]. The 1.2 kb *TLC1* RNA acts as a scaffold, providing separate binding sites for telomerase subunits Est1p and Est2p, the Sm protein complex, and the Ku heterodimer [11]. Association of the 7-member Sm complex is critical for RNA maturation [12], while Ku binding is important for nuclear retention of the RNA and efficient telomerase recruitment to telomeres [13]–[15]. Est2p, a reverse transcriptase [16], and *TLC1* RNA are sufficient for *in vitro* activity and are thus considered the catalytic core of the enzyme [17]. Both Est1p and Est3p are regulatory or accessory proteins since each is dispensable *in vitro* but required *in vivo* to maintain telomere length [7]–[9], [17]. The Est3p regulatory subunit is recruited to the complex through direct interactions with Est1p and Est2p, and stimulates telomerase activity *in vitro* [18], [19].

Est1p binds *TLC1* RNA via three secondary structural elements within sub-helix IVc: a pentanucleotide bulge, an adjacent internal loop, and a single-stranded region at the base of the sub-helix [20], [21]. In addition to its interaction with the RNA, Est1p is also important for the recruitment of telomerase to the telomere through a direct interaction with the telomeric single-stranded DNA binding protein, Cdc13p [22]–[26]. The Est1 protein undergoes proteasome-dependent cell cycle-regulated destruction in G1 phase, thereby preventing telomerase complex assembly during G1 phase when telomerase is not active at telomeres [27].

Protein destruction by the proteasome is regulated through the attachment of the small polypeptide ubiquitin to target molecules. Such ubiquitin-dependent protein degradation is accomplished through a multi-step process: the ubiquitin moiety is activated by an E1 activating enzyme, transferred to an E2 conjugating enzyme, and finally, covalently attached to lysine residues present within a target protein that is bound to an E3-ligase. Multiple rounds of this process result in polyubiquitinated proteins that are subsequently delivered to the 26S proteasome for degradation. Temporal coordination of ubiquitination and proteolysis of key regulatory proteins is critical for unidirectional progression of the cell cycle [28]. One of the well-studied poly-ubiquitinating E3 complexes with this role is the Anaphase Promoting Complex (APC).

The APC is a multi-subunit E3 ubiquitin ligase that is critical for transit through the cell cycle. Although the core subunits are constitutively expressed [29]–[31], APC functionality oscillates, exhibiting no activity in S and G2 phase, and high activity during mitosis and G1 phase [32]. The APC utilizes two evolutionarily conserved, WD40-domain containing activators, Cdc20p/Fizzy and Cdh1p/Hct1/Fizzy-related [33]–[35]. These activators bind directly to substrates via degradation motifs [36]–[39], the best characterized being the Destruction box (D-box: an arginine and leucine separated by any two amino acids, RxxL) and KEN-box [40]–[42]. The binding of these activators to the APC core particle is tightly regulated: Cdc20p associates when cyclin-dependent kinase (CDK/Cdc28p) activity is high in mitosis, while Cdh1p association is inhibited by phosphorylation and therefore occurs when CDK activity is low at the end of mitosis through G1 phase [43]–[45]. The direct binding of pseudosubstrate inhibitors and degradation of activator proteins also contribute to temporal regulation of APC activity [46]. APC^Cdc20p^ is critical during mitosis when specific recognition and subsequent destruction of the separase inhibitor (securin/Pds1p) results in cohesin cleavage, and thus sister-chromatid separation [35], [47]. APC^Cdh1p^ activity promotes exit from mitosis and ensures that CDK levels remain low, allowing for loading of replication origins with initiation proteins prior to the beginning of S phase, when CDK activity increases [46], [48], [49].

Est1p undergoes cell cycle-regulated degradation during G1 phase, thereby preventing Est3p recruitment and telomerase complex assembly [27]. Here we present evidence that Est1 protein levels oscillate during the cell cycle through an APC-dependent mechanism *in vivo*. Degradation requires three sequences in Est1p that match the D-box consensus, consistent with direct recognition of Est1p by the APC. However, recombinant Est1 protein is not degraded or ubiquitinated by the APC *in vitro*, suggesting that Est1p either lacks the necessary structure or modification(s) that influence APC recognition *in vivo* or is an indirect target of the APC. Because Est1p stimulates association of Est3p with the telomerase complex, these results shed light on the regulation of yeast telomerase biogenesis and demonstrate an additional connection between telomere maintenance and cell cycle regulation pathways.

## Results and Discussion

### Est1p is Stabilized in Early S phase

**Figure 1:**
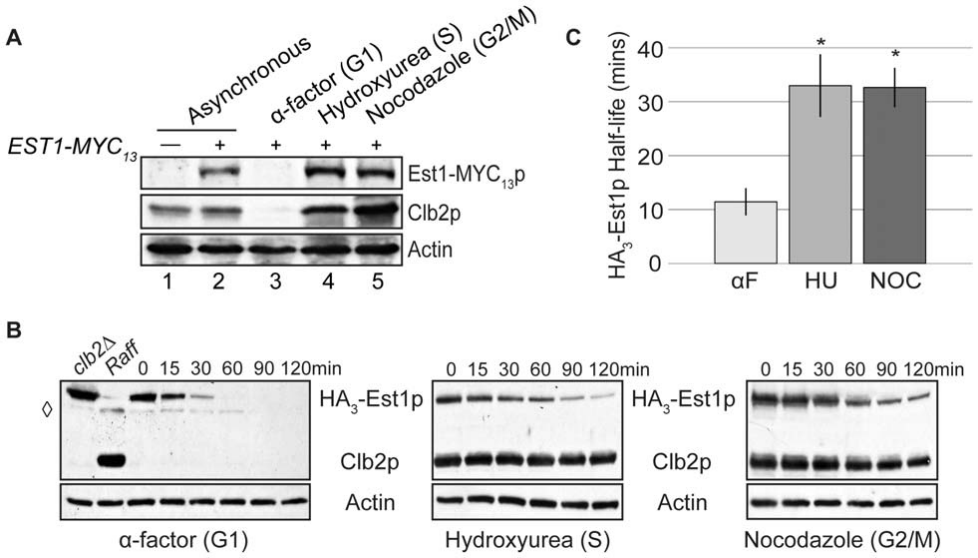
Est1p is unstable in G1 phase, but stable in early S and G2/M phases. (A) Endogenously expressed Est1p-MYC_13_p levels during cell cycle arrests. Strains YKF800 (untagged; lane 1) and YKF801 (*EST1-MYC_13_*; lanes 2–5) were grown asynchronously at 30°C to mid-log phase and then left untreated (asynchronous) or arrested by addition of α-factor, hydroxyurea, or nocodazole, as indicated. When 95% of the population was arrested, as monitored by the bud-index, cells were harvested. Whole-cell extract was prepared and western blotted using anti-MYC, anti-Clb2p, and anti-Actin antibodies, as indicated. (B) Half-life of HA_3_-Est1p during cell cycle arrests. Strain YKF802 containing plasmid pVL242RtoA (*P_GAL1_-HA_3_-EST1*) was grown asynchronously at 30°C to mid-log phase and arrested with α-factor, hydroxyurea, or nocodazole, as indicated. When 95% of the population was arrested, as monitored by the bud-index, expression of *HA_3_-EST1* was induced with addition of galactose and then subsequently repressed (after 1 hour) with glucose and cycloheximide (time 0). Samples from cells harvested at the indicated times were western blotted with anti-HA, anti-Clb2p and anti-Actin antibodies, as indicated. An induced asynchronous sample of strain YKF806+ pVL242RtoA (*clb2Δ*; left panel), served as a negative control for Clb2p and positive control for HA_3_-Est1p detection. An uninduced asynchronous sample of strain YKF802+ pVL242RtoA (Raff; left panel) served as a positive control for Clb2p detection and negative control for HA_3_-Est1p specificity. A non-specific background band is indicated by 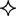. (C) Quantification of data shown in (B), as described in Materials and Methods. The calculated half-lives were averaged from independent biological replicates: αF (α-factor), n = 7; HU (hydroxyurea), n = 4; NOC (nocodazole), n = 4. Error bars are standard deviation from the mean. Both HU and NOC are statistically different from αF by two-tailed t-test (p-values 1.1×10^−5^ and 1.1×10^−6^, respectively) as denoted by *.

The telomerase recruitment protein, Est1p, undergoes degradation in G1 phase but not G2/M phase [27]. To more thoroughly examine the temporal regulation of Est1 protein levels, cells expressing MYC_13_-tagged *EST1* from its endogenous locus were arrested at three points in the cell cycle: G1 with the mating pheromone, alpha-factor; early S with the ribonucleotide reductase inhibitor, hydroxyurea; and late G2/M with the microtubule destabilizing agent, nocodazole. The efficiency of arrest was confirmed to be greater than 95% in each experiment by flow cytometry (Figure S1) and observation of bud index (data not shown). As expected, Est1-MYC_13_p was readily detected in whole-cell extract from asynchronously growing cells but not in the untagged control strain, indicating specificity of the MYC-antibody (Figure 1A, lanes 1 and 2). In agreement with previous observations [26], [27], endogenously expressed Est1-MYC_13_p was undetectable in G1 phase and abundant in G2/M-arrested cells (Figure 1A, compare lanes 3 and 5). Similar to G2/M-arrested cells, Est1-MYC_13_p was readily detected from early S-arrested cells (Figure 1A, lane 4), suggesting that Est1 protein levels increase as cells enter S phase.

Although *EST1* transcript levels are ∼3 fold lower in G1 phase than during G2/M [50], [51], we have previously shown that differential protein stability is an important factor determining Est1p levels during the cell cycle [27]. To examine the kinetics of Est1p degradation at different points in the cell cycle, protein half-life was determined using a standard promoter shut-off assay. Following a brief induction of *HA_3_-EST1* expression from the *GAL1*-promoter, both transcription and translation were inhibited and protein abundance was examined over time. As shown in Figure 1B, and in agreement with published work [27], HA_3_-Est1p was rapidly degraded during a G1 phase arrest, but was more stable during a G2/M phase arrest [27]. In accordance with the steady state protein levels (Figure 1A), over-expressed HA_3_-Est1p was also stable when cells were arrested in early S phase with hydroxyurea (Figure 1B, middle). Quantification of these assays confirmed a statistically significant increase in protein half-life during early S and G2/M phase as compared to G1 phase (Figure 1C; p-values = 1.1×10^−5^ and 1.1×10^−6^, respectively). Together, these results suggest that Est1p is rapidly degraded during G1 phase, stabilizes in early S phase, and remains stable through G2/M phase.

### Est1p is More Stable in G1 phase when APC Activity is Compromised

The pattern of Est1p degradation during the cell cycle is reminiscent of that observed for targets of the E3-ubiquitin ligase complex, APC. As a comparison, levels of the B-type cyclin Clb2p, a known APC substrate [52], [53], were monitored within the same extracts utilized for Est1p detection. As expected, Clb2p was undetectable in G1 and robustly detected in both S and G2/M arrested cells (Figure 1A and 1B). In addition to confirming the efficiency of cell cycle arrest, these results led us to hypothesize that Est1p degradation depends upon APC function.

**Figure 2:**
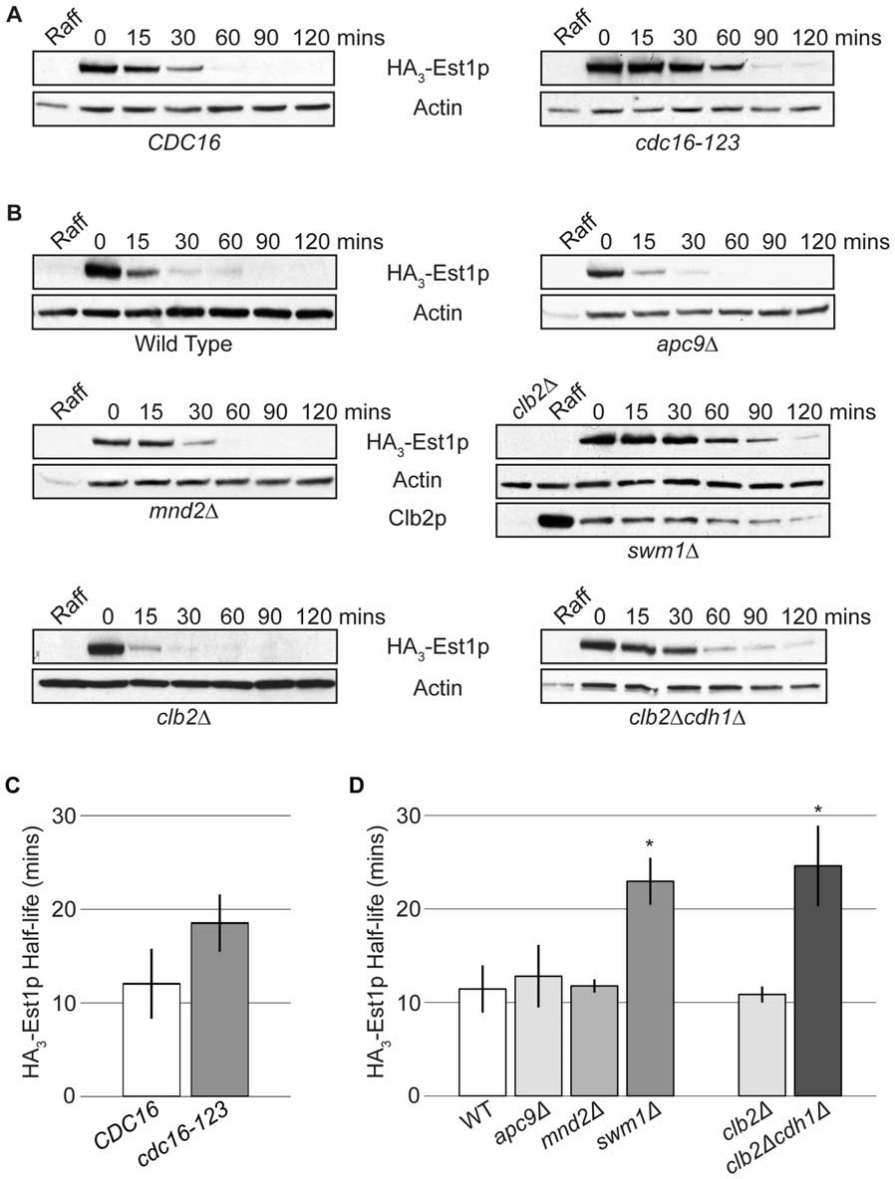
APC function is required for normal Est1p degradation during G1 phase. (A) HA_3_-Est1p stability increases when APC function is compromised. Western blots of Est1p stability assays from strain K4438 (*cdc16-123*) harboring pKF600 (*GAL1-HA_3_-EST1*) plus either a complementing vector pRS416-*CDC16* (labeled “*CDC16*”) or an empty vector pRS416 (labeled “*cdc16-123*”) were conducted as described in Materials and Methods. An uninduced sample (Raff) served as a negative control for HA_3_-Est1p specificity. (B) HA_3_-Est1p is stabilized in APC deletion mutants. Western blots of Est1p stability assays from strains YKF802 (Wild Type), YKF803 (*apc9Δ*), YKF804 (*mnd2Δ*), YKF805 (*swm1Δ*), YKF806 (*clb2Δ*) and YKF807 (*clb2Δcdh1Δ*) containing pVL242RtoA (*P_GAL1_-HA_3_-EST1*) were conducted as described in Materials and Methods. For YKF805 (*swm1Δ*), an uninduced asynchronous sample (Raff) served as a positive control for Clb2p detection and negative control for HA_3_-Est1p specificity, while an uninduced asynchronous sample of strain YKF806 (*clb2Δ*) served as a negative control for Clb2p detection. (C) Quantification of results shown in (A). Bars represent the average HA_3_-Est1p half-life from three independent biological replicates. Error bars are standard deviation of the mean (p-value = 0.08 by two-tailed t test). (D) Quantification of results shown in (B). Bars represent the average HA_3_-Est1p half-life from independent biological replicates: n = 3 for all strains except *clb2Δcdh1Δ*, where n = 4. Error bars are standard deviation from the mean. By two-tailed paired t-test, there is a significant difference between the control (WT) and *swm1Δ* (p-value 0.0002) but not between WT and *apc9Δ* (p-value 0.49) or *mnd2Δ* (p-value 0.83). There is a significant difference between the control (*clb2Δ*) and *clb2Δcdh1Δ* strains (p-value 0.003). Significant differences are denoted by *.

We monitored the degradation rate of over-expressed HA_3_-Est1p in alpha-factor arrested cells expressing the temperature-sensitive (ts) allele *cdc16-123*. This allele renders the APC non-functional at the restrictive temperature of 37°C and exhibits proteolysis defects with known APC substrates [52], [53]. A strain harboring the *cdc16-123* allele was transformed with a complementing *CEN* vector expressing wild-type *CDC16* under control of its endogenous promoter (denoted *CDC16*) or an empty-vector (denoted *cdc16-123*). Using the promoter shut-off assay described above, the average half-life of over-expressed HA_3_-Est1p was greater in the *cdc16-123* strain than in the complemented strain (Figure 2A and 2C) and trended toward significance with a p-value of 0.08. Therefore, we wanted to verify the relevance of this trend by examining other strains that compromise APC function *in vivo*.

Although APC activity is critical for cell viability, several subunits of this large E3 ubiquitin ligase are encoded by non-essential genes (*e.g.* Apc9p, Mnd2p, and Swm1p). Proteolysis of known APC substrates is minimally compromised in *apc9Δ* and *mnd2Δ* cells, suggesting that these two subunits exhibit substrate-specific effects or have minor contributions to full APC function. However, proteolysis of known APC substrates securin/Pds1p, Clb2p, Cdc5p, and Ase1p is decreased in *swm1Δ* cells, indicating a greater contribution to full APC activity [54], [55]. Using the promoter shut-off assay, the half-life of over-expressed HA_3_-Est1p was determined in *apc9Δ*, *mnd2Δ,* or *swm1Δ* cells arrested in G1 phase with alpha-factor (Figure 2B and 2D). HA_3_-Est1p was significantly more stable during G1 phase in *swm1Δ* cells than in wild-type cells (p-value = 0.0002), while the rate of degradation was unaffected by the deletion of either *apc9* or *mnd2* (p-values = 0.49 and 0.84, respectively). Endogenously expressed Clb2p was detected in the same *swm1Δ* samples, but was undetectable from *apc9Δ* or *mnd2Δ* samples (Figure 2B and data not shown), confirming the predicted phenotype of these strains. Thus, like known APC targets, normal Est1p degradation during G1 phase requires Swm1p function.

During G1 phase, the APC is associated with the activator protein Cdh1p. Like *SWM1*, *CDH1* is non-essential, most likely because securin and B-type cyclins, essential substrates of the APC, are sufficiently targeted by the mitotic activating factor, Cdc20p [56]. We examined the protein half-life of Est1p in *cdh1Δ* cells arrested in alpha-factor. Since deletion of *cdh1* results in cyclin accumulation that leads to bypass of the alpha-factor arrest [34], [48], [57], these analyses were performed in a *clb2Δ* background to prevent cells from moving into S phase. Consistent with the results obtained in *swm1Δ* cells, over-expressed HA_3_-Est1p was significantly stabilized in *clb2Δcdh1Δ* cells arrested in G1 phase (p-value = 0.003; Figure 2B and 2D, compare to *clb2Δ*). While clearly increased in comparison to the wild-type strain, the half-life of Est1p in alpha-factor arrested *swm1Δ* (T_1/2_ = 23+/−2.5 mins) or *clb2Δcdh1Δ* (T_1/2_ = 25+/−4.3 mins) strains was lower than that of the corresponding WT strain arrested with hydroxyurea (T_1/2_ = 33+/−5.8 mins; p-values 0.04 and 0.06, respectively) or nocodazole (T_1/2_ = 33+/−3.7 mins; p-values 0.01 and 0.03, respectively). These differences are consistent with the retention of partial APC activity in these viable strains. Collectively, these experiments support the hypothesis that the APC plays a role in the G1-specific degradation of Est1p.

### *CDH1* is Required for the Cell-cycle Oscillation of Est1 Protein Levels

The loss of Est1p during an alpha-factor arrest (Figure 1A) could be over-emphasized due to the artificial length of G1 phase. To confirm the kinetics with which Est1p levels fluctuate as cells enter and traverse an unperturbed G1 phase, we examined levels of endogenously expressed Est1-MYC_13_p after release of *cdc15-2* cells from mitotic arrest. *CDC15* encodes a protein kinase required for mitotic exit and incubation of *cdc15-2* cells at the restrictive temperature of 37°C results in cell cycle arrest in late anaphase/telophase [58]. Because CDK activity is elevated and the Cdc14p phosphatase is sequestered in the nucleolus and unable to dephosphorylate Cdh1p [43]–[45], [59], APC^Cdh1p^ is not active during the *cdc15-2* arrest. In contrast, the observation that *cdc15-2* arrested cells have separated chromosomes indicates that APC^Cdc20p^ is active and able to mediate Pds1p proteolysis prior to the arrest point. *cdc15-2* cells were incubated at the restrictive temperature until 95% of the population was arrested with the characteristic “dumbbell” morphology and then released from the arrest by shifting back to the permissive temperature of 23°C. Samples were harvested every 20 mins following release. Synchrony of the release was monitored by analysis of Clb2p levels, observation of the bud index (Figure S2), and flow cytometry (data not shown).

**Figure 3:**
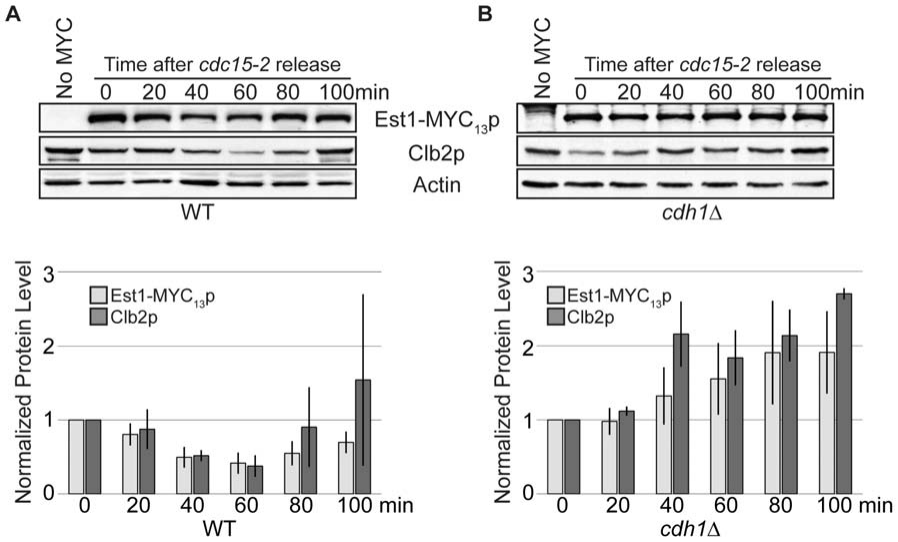
Cell-cycle oscillation of Est1p requires Cdh1p. (A) Est1 protein levels oscillate through the cell cycle. Strain YKF808 (*cdc15-2 EST1-MYC_13_)* was grown asynchronously at 23°C to mid-log phase and shifted to the restrictive temperature (37°C) for 3.5 hrs. When 95% of the cells were arrested, as monitored by bud-index (Figure S2), the culture was returned to the permissive temperature (23°C; time 0). Whole-cell extract was prepared from samples harvested every 20 mins following release and western blotted using anti-MYC, anti-Clb2p, and anti-Actin antibodies, as indicated. YCM191 (*cdc15-2*) served as the untagged (No MYC) control for Est1-MYC_13_p and was harvested following the 37°C incubation period. Est1-MYC_13_p and Clb2p intensity at each time were normalized to input (actin) and starting amount (time 0). Bars represent the average of four independent biological replicates for Est1-MYC_13_p (light) and Clb2p (dark); error bars are standard deviation of the mean. (B) Deletion of *CDH1* perturbs the oscillation of Est1-MYC_13_p through the cell cycle. Strain YKF809 (*cdc15-2 cdh1Δ EST1-MYC_13_*) was treated as in (A), except the bars represent the average of three independent biological replicates.

In agreement with published reports, endogenous Clb2p levels decreased upon release from the *cdc15-2* arrest [34], [60], [61], with the lowest point of expression at 40 to 60 mins (Figure 3A). Approximately 50% of cells show the first evidence of bud formation 60 mins after release (Figure S2), consistent with the interpretation that the trough of Clb2 expression corresponds to G1 phase. Examination of Est1-MYC_13_p levels within these same samples revealed a similar pattern; the lowest point of expression occurred 40–60 mins after release from the *cdc15-2* block (Figure 3A). Four independent biological replicates were done to demonstrate the reproducibility of this G1 phase decrease for both Clb2p and Est1-MYC_13_p (Figure 3A). The expression of both proteins at 40 and 60 mins after release was significantly decreased from the protein levels observed at the *cdc15-2* arrest (p-values for Clb2p: 6.0×10^−5^ and 1.7×10^−4^, respectively; p-values for Est1-MYC_13_p: 3.7×10^−4^ and 1.9×10^−4^, respectively).

Although the pattern with which Clb2p and Est1p declined in abundance after *cdc15-2* release was very similar, only Clb2p showed a large increase in expression at the end of the time course (100 mins) compared to the starting protein level. We attribute this behavior to the previous observation that a fraction of Clb2p undergoes APC^Cdc20p^-dependent degradation [61], which would be expected to have occurred prior to the *cdc15-2* arrest. Therefore, the starting protein levels observed for Clb2p at the *cdc15-2* arrest may already be partially reduced, with additional degradation attributable to APC^Cdh1p^ activity. The failure of Est1p to accumulate above the starting amount by the end of the time course suggests that Est1p may not undergo degradation in late mitosis, prior to the *cdc15-2* arrest point.

Since Cdh1p plays a role in Est1p degradation during G1 phase (Figure 2B and 2D), we hypothesized that deletion of this APC-activator would abrogate the protein oscillation pattern observed from cells released from a *cdc15-2* arrest. A *cdh1Δ cdc15-2* strain was incubated at restrictive temperature until >95% of the cells were arrested and then released by lowering the temperature. As monitored by both the bud index (Figure S2) and flow cytometry (data not shown), the cells proceeded into the next cell cycle similarly to wild type, with the emergence of small buds beginning at 60 mins after release. Consistent with published work, Clb2p levels no longer decreased during transit through G1 phase in *cdh1Δ* cells (Figure 3B; [34]). Importantly, Est1-MYC_13_p also did not exhibit a decline in protein levels as cells proceeded through G1 phase following release from the *cdc15-2* arrest (compare Figure 3A and 3B). We attribute the increase in Est1-MYC_13_p and Clb2p over their respective starting amounts (time 0) to result from the combination of lack of degradation and additional transcription/translation as cells exit the arrest [27], [50], [51], [62], [63]. Based on the preceding analysis of protein levels as cells exit mitosis and enter the following S phase, we conclude that Est1p likely undergoes proteolysis solely during G1 phase, stabilizes as cells transit through S phase, and remains stable through mitosis. Furthermore, APC^Cdh1p^ is the primary regulator of the G1 phase-specific proteolysis of Est1p.

### Mutation of *cis*-acting Sequences Stabilizes Est1p in G1 Phase

**Figure 4:**
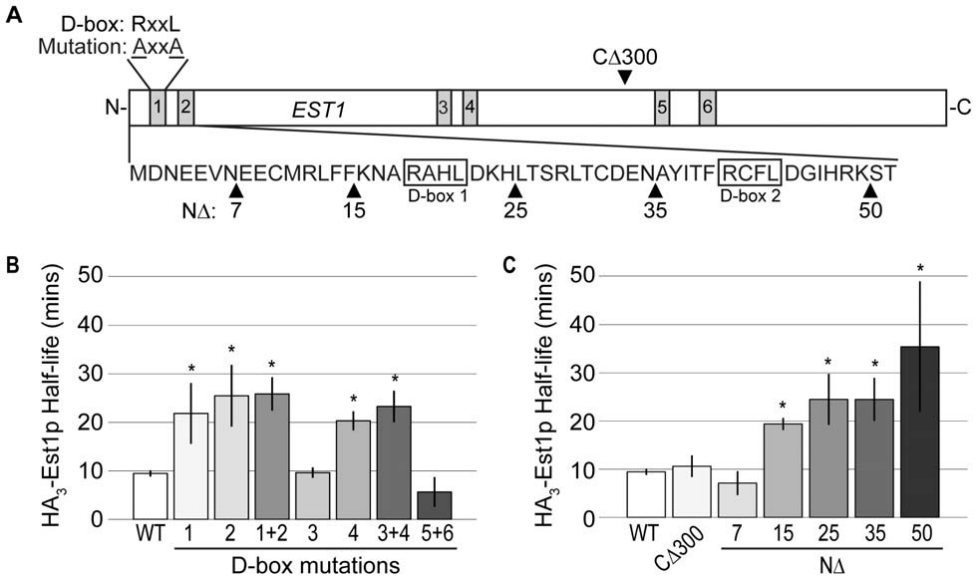
Est1p degradation in G1 phase requires three destruction boxes (D-boxes). (A) Schematic of *EST1* shown to scale. *EST1* contains six putative D-boxes with sequence RxxL (boxes labeled 1–6). Deletion of the C-terminal 300 amino acids (CΔ300) results in a truncated protein that removes putative D-boxes 5 and 6. The N-terminal 52 amino acids are shown, with putative D-boxes 1 and 2 outlined. Upward pointing black triangles represent the position of the indicated N-terminal deletion. (B) D-boxes 1, 2, and 4 contribute to Est1p degradation. YKF802 containing pKF600 (*GAL1-HA_3_-EST1*) plasmids expressing either wild-type *EST1* (WT) or the D-box (DB) mutated (RxxL to AxxA) *est1* alleles indicated were treated as in Figure 1B, except strains were arrested with α-factor. Bars represent the average HA_3_-Est1p half-life for three independent biological replicates; error bars are the standard deviation of the mean. Using a two-tailed t-test, there is no significant difference from WT for D-box 3 (p-value 0.833) or D-boxes 5+6 (p-value 0.104). D-box 1 (p-value 0.027), D-box 2 (p-value 0.012), D-boxes 1+2 (p-value 0.001), D-box 4 (p-value 0.001) and D-boxes 3+4 (p-value 0.002) are significantly different than WT, denoted by *. (C) Deletion of D-box 1 or 2 stabilizes Est1p during G1 phase. YKF802 containing pKF600 plasmids expressing either wild-type *EST1* (WT) or the *est1* deletion variants indicated (CΔ300, NΔ7, NΔ15, NΔ25, NΔ35 or NΔ50) were treated as in (A). Bars represent the average HA_3_-Est1p half-life for independent biological replicates: n = 3 for each variant except NΔ50, where n = 4. Error bars are standard deviation from the mean; significance is denoted by *. By a two-tailed t-test, there is no significant difference between WT and CΔ300 (p-value 0.445) or NΔ7 (p-values 0.188). The half-lives observed for NΔ15 (p-value 0.0003), NΔ25 (p-value 0.008), NΔ35 (p-value 0.005) and NΔ50 (p-value 0.02) are significantly different from WT.

The data presented thus far demonstrate that the APC influences the G1 phase-specific degradation of Est1p. However, these experiments do not address whether Est1p is a direct substrate of the APC. Substrates of the APC are recognized through specific degron motifs such as the Destruction box (D-box: sequence RxxL) and KEN box [40]–[42]. If Est1p is a direct target of APC^Cdh1p^, we would predict *EST1* to encode specific degron(s) important for the recognition and subsequent proteolysis of Est1p. Examination of the amino acid sequence of Est1p revealed the presence of six putative D-boxes positioned in pairs throughout the protein (Figure 4A). To test if any of these putative D-boxes has a role in Est1p degradation during G1 phase, we mutated the important arginine (R) and leucine (L) residues of each consensus sequence to alanine (A) and determined protein half-life using a promoter shut-off assay in cells arrested with alpha-factor. Individual mutation of putative D-boxes 1, 2, and 4 stabilized the protein during G1 phase while no significant increase in half-life was observed upon mutation of putative D-boxes 3, 5, or 6 (Figure 4B and Figure S3A; data not shown). Because the degron motifs occurred in pairs, we also asked whether mutating each pair of putative D-boxes (1+2, 3+4, or 5+6) would further inhibit proteolysis. Consistent with the single D-box data, combined mutation of 1+2 or 3+4 resulted in stabilization of the protein, but the effect was not additive. No stabilization was observed upon mutation of D boxes 5+6 (Figure 4B and Figure S3A). The extent to which the half-life increased for either the single or combined mutations was not statistically different from the half-life observed during a nocodazole (G2/M phase) arrest, suggesting that the loss of a single D-box motif is sufficient to stabilize the protein in G1 phase.

To corroborate the results obtained with the specific point mutations described above, we also monitored the half-life of several deletion variants of Est1p. Deletion of the C-terminal 300 amino acids (denoted CΔ300 in Figure 4A) removes putative D-boxes 5 and 6, previously shown not to contribute to Est1p degradation during G1 phase (Figure 4B). Consistent with that conclusion, the half-life of Est1p^CΔ300^ remained unchanged compared to the full-length protein, suggesting that putative D-boxes 5 and 6 are not degron motifs (Figure 4C and Figure S3B). A larger C-terminal deletion (CΔ500) did not express well; we were therefore unable to examine the stability of an Est1p peptide containing only D-boxes 1 and 2 (data not shown). We next created systematic deletions from the N-terminus of *EST1* to assess the influence of D-boxes 1 and 2 on Est1p degradation. The N-terminal boundaries of D-boxes 1 and 2 are located at amino acid 19 and 41, respectively (Figure 4A). We constructed five N-terminal deletions: *est1^NΔ7^*, *est1^NΔ15^*, *est1^NΔ25^*, *est1^NΔ35^*, and *est1^NΔ50^*. Examination of the protein half-life via promoter shut-off assay revealed no stabilization with the smallest deletion (Est1p^NΔ7^), but the half-lives of Est1p^NΔ25^, Est1p^NΔ35^, and Est1p^NΔ50^ were increased (Figure 4C and Figure S3B). Again consistent with the lack of additivity previously observed, loss of putative D-box 1 (Est1p^NΔ25^ and Est1p^NΔ35^) was equivalent in effect to loss of both putative D-boxes 1 and 2 (Est1p^NΔ50^). The extent of stabilization observed in the deletion variants was similar to that observed with the point mutations (compare Figure 4B and 4C). Est1p^NΔ15^ was more stable than the full-length protein even though no portion of a predicted D-box was deleted with this construct (Figure 4C and Figure S3B). Since this deletion retains only 3 amino acids N-terminal to the beginning of D-box 1, we attribute this stabilization to misfolding of D-box 1 and disrupted recognition by APC^Cdh1p^. However, it is possible that a novel degron motif exists between amino acids 7 and 15. These results are consistent with Est1p being a direct substrate of the APC and suggest that *EST1* encodes three degron motifs (D-boxes 1, 2, and 4) important for recognition and subsequent degradation during G1 phase.

### Neither Proteolysis Nor Ubiquitination of Recombinant Est1p by the APC Occurs *in vitro*

**Figure 5:**
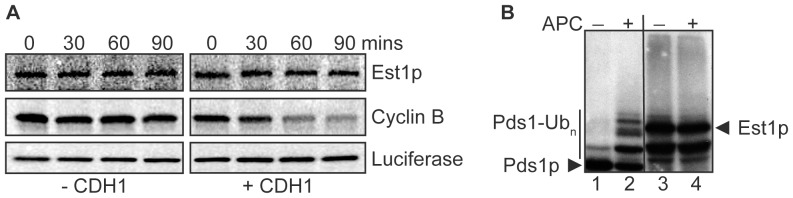
Est1p is not a target of the APC *in vitro.* (A) Est1p is not degraded by the APC *in vitro*. *X. laevis* egg extract (− CDH1) was activated by the addition of *in vitro* transcribed human Cdh1 to obtain APC-activated extract (+ CDH1). ^35^S-labeled substrate proteins (*S. cerevisiae* Est1p, *D. melanogaster* Cyclin B, or firefly luciferase) were incubated with either inactive (− CDH1) or activated extract (+ CDH1) as described in Materials and Methods. Samples were removed at the indicated times, separated by gel-electrophoresis and exposed to a phosphor-imager screen. (B) Est1p is not ubiquitinated *in vitro*. ^35^S-labeled substrates (*S. cerevisiae* Est1p and Pds1p) were incubated with Ubc4p (E2 ligase), recombinant *S. cerevisiae* Cdh1p, and methylated-ubiquitin in the absence (−; lanes 1 and 3) or presence (+; lanes 2 and 4) of purified *S. cerevisiae* APC complexes. Reactions were separated by gel electrophoresis and detected by autoradiography film. Black arrows indicate the unmodified protein. The vertical line indicates the region where ubiquitin-conjugated forms of Pds1p migrate.

The analyses described above suggest that Est1p undergoes G1-specific degradation that is dependent upon direct recognition of degron motifs within the protein by APC^Cdh1p^. To examine the direct effect of APC^Cdh1p^ on Est1p, we monitored degradation of recombinant Est1p using *Xenopus laevis* egg extracts either without (APC inactive) or with (APC active) human Cdh1 supplementation. Recombinant *Drosophila* cyclin B served as a positive control for APC-mediated degradation, while firefly luciferase served as the negative control. As expected, luciferase remained stable while cyclin B was efficiently degraded in the presence of Cdh1 (Figure 5A). However, there was no observed degradation of recombinant Est1p when Cdh1 was added (Figure 5A).

To eliminate the possibility of cross-species incompatibility, we also tested whether Est1p is ubiquitinated by APC^Cdh1p^ *in vitro* when all components of the assay are either purified from *S. cerevisiae* or are recombinant proteins of *S. cerevisiae* origin. ^35^S-labeled substrates synthesized in rabbit reticulocyte lysate (RRL) were incubated with methylated-ubiquitin and recombinant Cdh1p in the presence (+) or absence (−) of purified *S. cerevisiae* APC complexes (Figure 5B). Methylated-ubiquitin prevents poly-ubiquitin chain formation; thus, substrate ubiquitination results in two observable changes: 1) loss of signal corresponding to the unmodified protein and 2) appearance of a ladder of higher molecular weight bands indicative of covalent attachment of a single ubiquitin moiety to individual lysines. *S. cerevisiae* securin/Pds1p, previously shown to undergo APC^Cdh1p^-dependent ubiquitination *in vitro* [64], served as a positive control. As expected, Pds1p was ubiquitinated in a manner dependent upon addition of both purified APC and recombinant Cdh1p [indicated by the ladder of higher molecular weight species and loss of the unmodified signal (Figure 5B, lane 2)]. In contrast, Est1p was not detectably ubiquitinated in this assay (Figure 5B, compare lanes 3 and 4).

Experiments designed to detect substrate ubiquitination *in vivo* are challenging because the ubiquitinated forms represent a small fraction of the total protein, are rapidly degraded by the proteasome, and are acted on by deubiquitinating enzymes (Dubs). Despite using techniques designed to limit these concerns [65], [66], we have not detected ubiquitination of overexpressed Est1p *in vivo* (data not shown). However, these analyses have not been exhaustive and do not rule out the possibility that a critical pool of Est1p undergoes ubiquitination *in vivo*.

The lack of APC^Cdh1p^-dependent ubiquitination or degradation of Est1p *in vitro* using two different assays contrasts with our identification of degradation motifs in Est1p that resemble those utilized by APC^Cdh1p^ in other substrates and that are required for Est1p degradation during G1 phase (Figure 4). One possibility is that modifications of Est1p influence recognition by APC^Cdh1p^ *in vivo* and that these modification(s) are not appropriately mimicked upon expression of Est1p in RRL. Although much of the regulation of APC-mediated degradation occurs through direct modulation of APC activity, post-translational modification of substrate molecules has been found to affect recognition by the APC in several cases including Cdc6, securin, and Aurora A [46], [67]–[70]. While it was recently reported that Est1p is not detectibly phosphorylated *in vivo* [18], the presence of other post-translation modifications has not been addressed. We also cannot exclude the possibility that recombinant Est1p is mis-folded, precluding recognition by APC^Cdh1p^ in the *in vitro* assays.

An alternate possibility is that the amino acids required for Est1p degradation *in vivo* (Figure 4) do not mediate direct interaction with APC^Cdh1p^, but are instead required for recognition by a currently unidentified ubiquitin ligase or protease. Because our results provide strong evidence that Est1p degradation depends upon APC^Cdh1p^ function (Figures 2 and 3), we would need to postulate that the effect of the APC is indirect. For example, Est1p may be targeted for degradation via a mechanism that itself is under positive regulation by the APC, reminiscent of cohesin cleavage by separase after Pds1/securin degradation via APC^Cdc20p^ [71].

Previous work has shown that Est1p regulates the assembly of the telomerase complex *in vivo*. However, even in the presence of abundant Est1 protein and telomerase complex assembly, telomerase is unable to elongate telomeres during G1 phase [27]. This observation suggests that additional regulatory mechanisms prevent inappropriate telomerase activity. A role for the Rif2 protein in G1-specific telomerase inhibition was recently reported [72]. However, these results do not rule out an additional regulatory role for Est1p degradation during G1 phase. In this light, it is intriguing that all of the D-box stabilizing mutations (Figure 4) cause telomere shortening when expressed under control of the endogenous promoter in *est1Δ* cells (Figure S4). While this observation suggests that the stabilization of Est1p during G1 phase may be deleterious, we cannot rule out the possibility that the mutations affect other aspects of Est1p function.

In summary, our *in vivo* results are most consistent with a model in which Est1p levels oscillate through the cell cycle, undergoing G1-specific degradation that is dependent upon APC^Cdh1p^-mediated recognition of specific degron motifs within the protein. Reduced Est1p levels during G1 phase are in turn predicted to restrict the assembly of the active telomerase complex [27]. Although we cannot rule out misfolding of the recombinant protein as an explanation for the lack of Est1p degradation *in vitro*, these results raise the intriguing possibility that additional regulatory events modulate Est1p abundance in a manner that depends upon APC function.

## Materials and Methods

### Ethics Statement

All work with *Xenopus laevis* was approved by the Institutional Animal Care and Use Committee (IACUC) at Vanderbilt University Medical Center (protocol #M/07/143) and was carried out in accordance with their policies and guidelines. *Xenopus laevis* were maintained by the Division of Animal Care (DAC) at Vanderbilt University’s Animal Care Facility, which provides both veterinary and husbandry services. Animals were monitored on a daily basis by the DAC for signs of morbidity (e.g. lethargy, open sores, and excessive skin shedding). Animals with these symptoms were subsequently euthanized by anaesthetic overdose with 0.05% Benzocaine absorbed through the skin, consistent with recommendations from the Panel on Euthanasia of the American Veterinary Medical Association.

### Yeast Strains and Plasmids

*S. cerevisiae* strains used in this study are summarized in Table S1. All gene disruptions were created using PCR-mediated gene disruption [73]; primer sequences are available upon request. The *bar1Δ::hisG* in K1534 [48]was replaced by amplification of the *bar1Δ::kanMX4* cassette from the yeast knockout collection [74] (Open Biosystems) to yield YKF800. The *bar1Δ::hisG* of K1534, MAY6810 and MAY6812 [53] was replaced using the *hphMX4* cassette from pAG32 [75] to yield YKF802, YKF806, and YKF807, respectively. YKF803, YKF804, and YKF805 were constructed by amplification of the *apc9*, *mnd2* and *swm1* gene disruption cassettes (*xxx::kanMX4*) from the yeast knockout collection and integrated into YKF802. A MYC_13_ epitope tag was incorporated at the C terminus of the endogenous *EST1* locus using plasmid pRS416-*EST1-MYC_13_-hphNT1* [derived from pFA6a-13MYC-kanMX6 [76], pYM16 (*hphNT1*) [77] (Euroscarf), and pRS416 [78]; details of plasmid construction available by request]. Digestion of this plasmid with *Sac*I and *Kpn*I yielded a linear DNA molecule containing homology upstream and downstream of the *EST1* chromosomal locus to allow one-step gene replacement. Transformation of this fragment into strains YKF800 and YCM191 yielded YKF801 and YKF808, respectively. YKF809 was created by PCR-amplification of the *cdh1Δ::KAN^R^* allele from MAY6812 and introduction into YKF808 by one-step gene replacement. To yield YKF810, the endogenous *EST1* locus was deleted in YKF802 by PCR-based gene deletion using plasmid pFA6a-kanMX6 [76].

Plasmids used in this study are summarized in Table S2. Plasmid pKF600 is derived from pVL242RtoA [27] and differs by the arrangement of restriction sites to facilitate cloning of mutant alleles. Individual D-box mutations (RxxL → AxxA) were constructed by site-directed mutagenesis using the SOEing method [79] and cloned into pKF600 to yield the indicated pKF600-DB plasmids. Simultaneous mutation of D-boxes 1+2, 3+4, or 5+6 was achieved by site-directed mutagenesis using a single mutant plasmid as the template in the PCR reaction. Deletion alleles of *EST1* were created by PCR amplification of a portion of *EST1* followed by insertion into pKF600. Plasmid pRS416-EST1 was created by PCR amplifying the *EST1* upstream promoter region, open reading frame (ORF), and downstream terminator region and cloning into pRS416. Mutant *est1* alleles were introduced by subcloning from the pKF600 vector series, to yield the indicated pRS416-DB and NΔ plasmids. The *CDC16* complementing plasmid was created in two steps: one primer pair amplified the promoter region and first-half of the *CDC16* ORF while a second primer pair amplified the second-half of the *CDC16* ORF and terminator. These two fragments were sequentially cloned into pRS416 to yield the complementing vector, pRS416-CDC16. To create plasmid pKF601, the *EST1* ORF was PCR-amplified and cloned into pCS2FA2R [derivative of pCS2; gift from Laurie Lee] at restriction sites *Fse*I and *Asc*I. The *EST1* ORF was PCR-amplified and cloned into pcDNA3.1-Hygro (Invitrogen) at restriction sites *Bam*HI and *Xba*I to yield pKF602.

### Determination of Est1p Steady-state Levels During Cell Cycle Arrest

Strains YKF800 (*bar1Δ*) and YKF801 (*bar1Δ EST1-MYC_13_*) were grown asynchronously at 30°C to OD_600_ ≈ 0.5 and either left untreated (asynchronous) or treated with α-factor (0.5 µM final concentration; Zymo Research), hydroxyurea (15 mg/ml) final concentration; Sigma Aldrich), or nocodazole (10 mg/ml nocodazole in DMSO to a final concentration of 10 µg/ml; Sigma Aldrich) for a minimum of 2.5 hrs. When 95% of the population exhibited the characteristic morphologies, cells were harvested and whole-cell extract prepared as described [80]; protein concentrations were determined by Bradford assay (Bio-Rad). Equal amount of protein extract (100–150 µg) were separated by 10% Tris-Glycine (Bio-Rad) and 7% NuPAGE Bis-Tris (Invitrogen) gels and transferred to Hybond P (GE Healthcare). Each membrane was blocked with 5% milk/phosphate-buffered saline pH 7.4 with 0.05% Tween (PBS-T) followed by incubation with primary antibodies overnight at 4°C. Antibody dilutions were as follows: Clb2-1∶6000 dilution rabbit polyclonal y-180 (Santa Cruz); Actin-1∶1200 goat polyclonal C-11 (Santa Cruz); 1∶1000 mouse monoclonal mAbcam8224 (Abcam); and MYC-1∶333 murine monoclonal Ab.1 (OP10L, EMD Biosciences). Bis-Tris gels were utilized for Est1-MYC_13_p detection because the protein co-migrated with a background band that could not be resolved using the 10% Tris-glycine gels. Secondary antibodies were 1∶10000 dilutions of peroxidase-conjugated goat anti-mouse [Millipore], goat anti-rabbit [Millipore], and donkey anti-goat [sc-2020; Santa Cruz]. ECL Plus Western Blotting Detection system (GE Healthcare) was used for detection.

For flow cytometry analysis, cells were treated as described in [81], except that samples were digested overnight with RNase A at 37°C instead of pepsin. Fluorescence and light scattering were monitored for 10,000 cells using a 5-laser BD LSRII. To eliminate any size-bias, samples were not gated and all events are plotted in the histograms presented.

### Over-expressed Sst1p Stability Assays and Half-life Quantification

Strains YKF802 (wild type), YKF803 (*apc9Δ*), YKF804 (*mnd2Δ*), YKF805 (*swm1Δ*) containing plasmid pVL242RtoA (*GAL1-HA_3_-EST1*) or variants of pKF600 (*GAL1-HA_3_-EST1*: WT; DB1; DB2; DB1+2; DB3; DB4; DB3+4; DB5+6; CΔ300; NΔ7; NΔ15; NΔ25; NΔ35; NΔ50) were assayed as described in [27], except using hydroxyurea (15 mg/ml final concentration; Sigma Aldrich) where indicated. For temperature-sensitive experiments, K4438 (*cdc16-123*) containing plasmid pKF600 and either a complementing vector (pRS416-*CDC16*) or empty vector (pRS416) were assayed as in [27], except for growth in 2% (w/v) raffinose media lacking leucine and uracil (-Leu -Ura) and a shift to the restrictive temperature (37°C) at the time of galactose addition. Samples were separated on 10% tris-glycine SDS-PAGE gels (Bio-Rad) and transferred to Hybond P (GE Healthcare). Membranes were blocked with 5% milk/phosphate-buffered saline pH 7.4 with 0.05% Tween (PBS-T) followed by incubation with primary antibodies (HA: 1∶500 dilution [murine monoclonal HA.11; Covance]; Clb2 and Actin, as above) overnight at 4°C. Secondary antibodies and detection system are as described above. For half-life determination, the signal obtained for HA_3_-Est1p at each time point was corrected for input (actin), normalized to the starting amount (time 0), base-*e* log-transformed, and plotted against time. The slope was determined using a linear best-fit line and used to calculate the half-life by T_1/2_ = ln(2)/slope, as described in [82].

### *cdc15-2* Block and Release

1000 ml cultures of strains YKF808 (*cdc15-2 EST1-MYC_13_*) and YKF809 (*cdc15-2 cdh1Δ EST1-MYC_13_*) were grown asynchronously at 23°C to OD_600_ ≈ 0.4 and then shifted to the restrictive temperature (37°C) in an air incubator for 3.5 hrs or until >95% of the population was arrested in mitosis, as determined by observation of the bud index. Cultures were released from the *cdc15-2* arrest by rapid cooling in an ice water bath to 23°C (time 0) and returned to a 23°C air incubator for the remainder of the experiment. Samples (125 ml) were harvested at 20 min intervals following release and whole-cell extract was prepared as described [80]. Synchrony of the release was monitored by bud-index and flow cytometry. YCM191 (untagged; *cdc15-2*) was grown as above and harvested following incubation at 37°C. Equal amounts of protein (120 µg), as determined by Bradford assay (Bio-Rad), were analyzed by Western blotting as described above. Est1-MYC_13_p and Clb2p signal intensity at each time point was normalized to protein input (actin) and starting amount (time 0). Samples from each assay were analyzed by Western blot two to three times and the quantified results were averaged to give yield a value for that independent assay. Averages determined from the independent biological replicates (WT = 4, *cdh1Δ* = 3) were subsequently averaged to yield the values reported in Figure 3. Standard deviation of the mean was determined across the independent biological replicates.

### *In vitro* Assays of Est1p Stability/ubiquitination

*Xenopus laevis* egg extracts were prepared in a manner similar to that previously described [42], [83]. Briefly, eggs from Human chorionic gonadotropin (HCG)-injected, pregnant mare serum gonadotropin (PMSG)-primed frogs were collected and washed in 1× Marc’s modified ringer (MMR) solution. Eggs were dejellied with 2% cysteine and then washed into extract buffer (XB) and XB containing protease inhibitors. A 30 sec packing spin at 1000 rpm at 2°C was performed, followed by a crushing spin at 13,000 rpm for 5 min at 2°C. The cytoplasmic layer was collected and subjected to a clarifying spin also at 13,000 rpm for 5 min at 2°C. The clarified cytoplasmic layer was collected. After addition of protease inhibitors, energy mix [83] and cytochalasin B, extracts were either frozen in liquid nitrogen (− CDH1) or activated with addition of *in vitro* transcribed human Cdh1-MYC_6_ RNA (+ CDH1) for 2 hrs at room temperature prior to flash freezing, and stored in liquid nitrogen. An anti-MYC Western blot confirmed successful translation of Cdh1-MYC_6_ RNA. ^35^S-labeled substrates (*S. cerevisiae* Est1p, *Drosophila* Cyclin B, and firefly luciferase) were produced using the TNT Sp6 Quick coupled *in vitro* transcription/translation (IVT) kit (Promega). Additional Cdh1-MYC_6_ protein was produced using the TNT Sp6 High-Yield Wheat Germ Protein Expression System (Promega), added (2 µL) to thawed+CDH1 extract, and incubated at room temperature for 15 mins. Inactive (− CDH1) and active (+ CDH1) extracts (10 µL) were incubated at room temperature with 1–2 µL recombinant substrates, energy mix, and ubiquitin (Sigma). Samples (2 µL) were taken at 0, 30, 60 and 90 mins and frozen in liquid nitrogen. Samples were separated on 10% Tris-glycine (Bio-Rad) SDS-PAGE gels, fixed, dried and exposed to a phosphor-imager screen.

APC/C ubiquitination assays were adopted and modified from [84]. ^35^S-labeled substrates and unlabeled *S. cerevisiae* Cdh1 were prepared using TNT T7 Quick coupled *in vitro* transcription/translation (IVT) (Promega). Each ubiquitination reaction contained approximately 10 ng of APC/C, 1 µl of ^35^S-labeled substrate, and 2 µl of Cdh1 in a 10 µl reaction volume with 40 mM Tris-HCl pH 7.5, 10 mM MgCl_2_, 0.6 mM DTT, 2.7 mM ATP, 6.6 µg of methyl-ubiquitin, 500 ng of Ubc4, 200 ng of ubiquitin aldehyde (Enzo Life Science), 2 mM LLnL (N-acetyl-Leu-Leu-Norleu-aldehyde; Sigma). Reactions were incubated at room temperature for 60 mins and were analyzed by 8% SDS-PAGE. Gels were fixed and stained with Coomassie Blue followed by drying and exposure to BioMax MR Film (Kodak).

### Southern Blotting

Strain YKF810 (*est1Δ*) containing pRS416-EST1 was grown in non-selective media, plated on 5-fluroorotic acid (5-FoA) to select for loss of the complementing plasmid, and then transformed using the standard lithium acetate method with an empty vector (pRS416), complementing plasmid (pRS416-EST1), or variants of pRS416 expressing mutant *est1* alleles (DB1; DB2; DB1+2; DB3; DB4; DB3+4; NΔ15; NΔ50). Independent transformants were restreaked three times on selective media and then grown in liquid culture. DNA was extracted by glass bead lysis [85], digested with *Xho*I, and Southern blotted as described in [86].

## Supporting Information

Figure S1 Flow cytometry of arrested cells. Example of the typical flow cytometry histograms resulting from *S. cerevisiae* strains used in this study left untreated (asynchronous; Asyn.) or arrested as indicated. The profile of hydroxyurea-blocked cells is nearly indistinguishable from that observed upon treatment with α-factor, consistent with an early S phase arrest in the vast majority of cells.
(TIF)

Figure S2 Cells released from the *cdc15-2* arrest proceed synchronously into the next cell cycle. Budding index of cells collected at the indicated times after release from the *cdc15-2* arrest (Figure 3). Results are from a single WT (light) and *cdh1Δ* (dark) assay and indicate the percentage of cells with visible buds. This result is representative of the pattern observed from the *cdc15-2* arrest and release assays.
(TIF)

Figure S3 Est1p degradation in G1 phase depends upon specific degron motifs. (A) Western blots of Est1p stability assays from strain YKF802 containing pKF600 (*GAL1-HA_3_-EST1*) plasmids expressing the D-box (DB) mutated (RxxL to AxxA) *est1* alleles indicated (DB1; DB2; DB1+2; DB3; DB4; DB3+4; DB5+6), treated as in Figure 1B (α-factor). (B) Strain YKF802 containing pKF600 plasmids expressing the deletion variants indicated (CΔ300, NΔ7, NΔ15, NΔ25, NΔ35 or NΔ50) were treated as in (A). Results are quantified in Figure 4.
(TIF)

Figure S4 Stabilized alleles of Est1p fail to complement an *est1* deletion. Independent isolates from strain YKF810 (*est1Δ*) harboring plasmids pRS416 (empty vector: ev), pRS416-*EST1* (EST1), or the *est1* alleles indicated (DB1; DB2; DB1+2; DB3; DB4; DB3+4, NΔ15; NΔ50) were propagated for >100 generations. DNA was extracted, digested with *Xho*I, Southern blotted, and probed with a randomly labeled telomeric DNA probe. Y′-elements and telomere fragments from Y′-containing chromosomes are indicated. Positions of molecular weight markers (M) are indicated in kilobases (kb). Alleles partially compromised for function have telomere fragments that are shorter than the wild-type control while severely compromised alleles result in the formation of telomerase-negative survivors characterized by Y′-element amplification and/or heterogeneous telomere length (smears throughout the lane).
(TIF)

Table S1 *S. cerevisiae* strains used in this study.
(DOCX)

Table S2 Plasmids used in this study.
(DOCX)
