# Supplementary figures and images for: The Anaphase Promoting Complex Contributes to the Degradation of the S. cerevisiae Telomerase Recruitment Subunit Est1p
Source: PLoS One. 2013 Jan 25;8(1):e55055. doi: 10.1371/journal.pone.0055055 (PMC3555863; doi:10.1371/journal.pone.0055055)

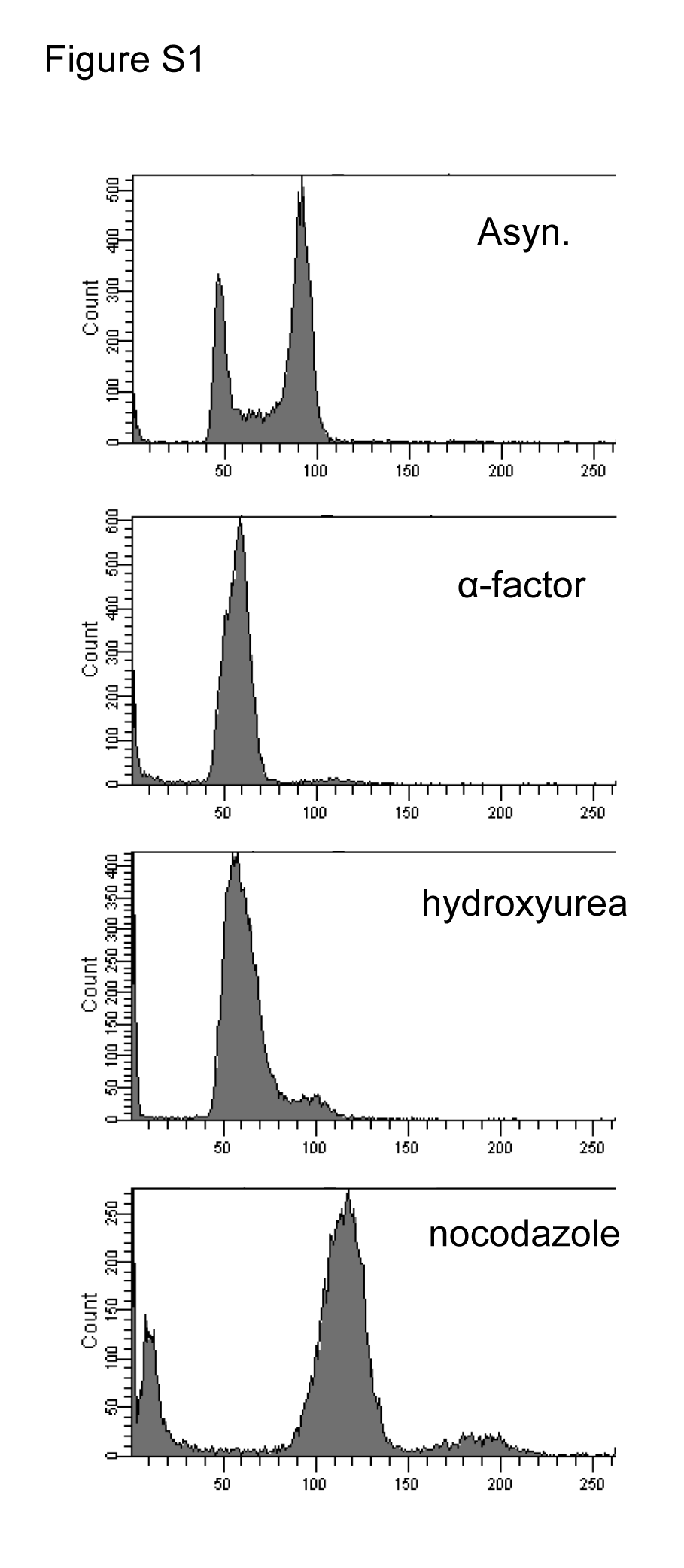

Supplement: Figure S1 — Flow cytometry of arrested cells. Example of the typical flow cytometry histograms resulting from S. cerevisiae strains used in this study left untreated (asynchronous; Asyn.) or arrested as indicated. The profile of hydroxyurea-blocked cells is nearly indistinguishable from that observed upon treatment with α-factor, consistent with an early S phase arrest in the vast majority of cells. (TIF) [file pone.0055055.s001.tif]

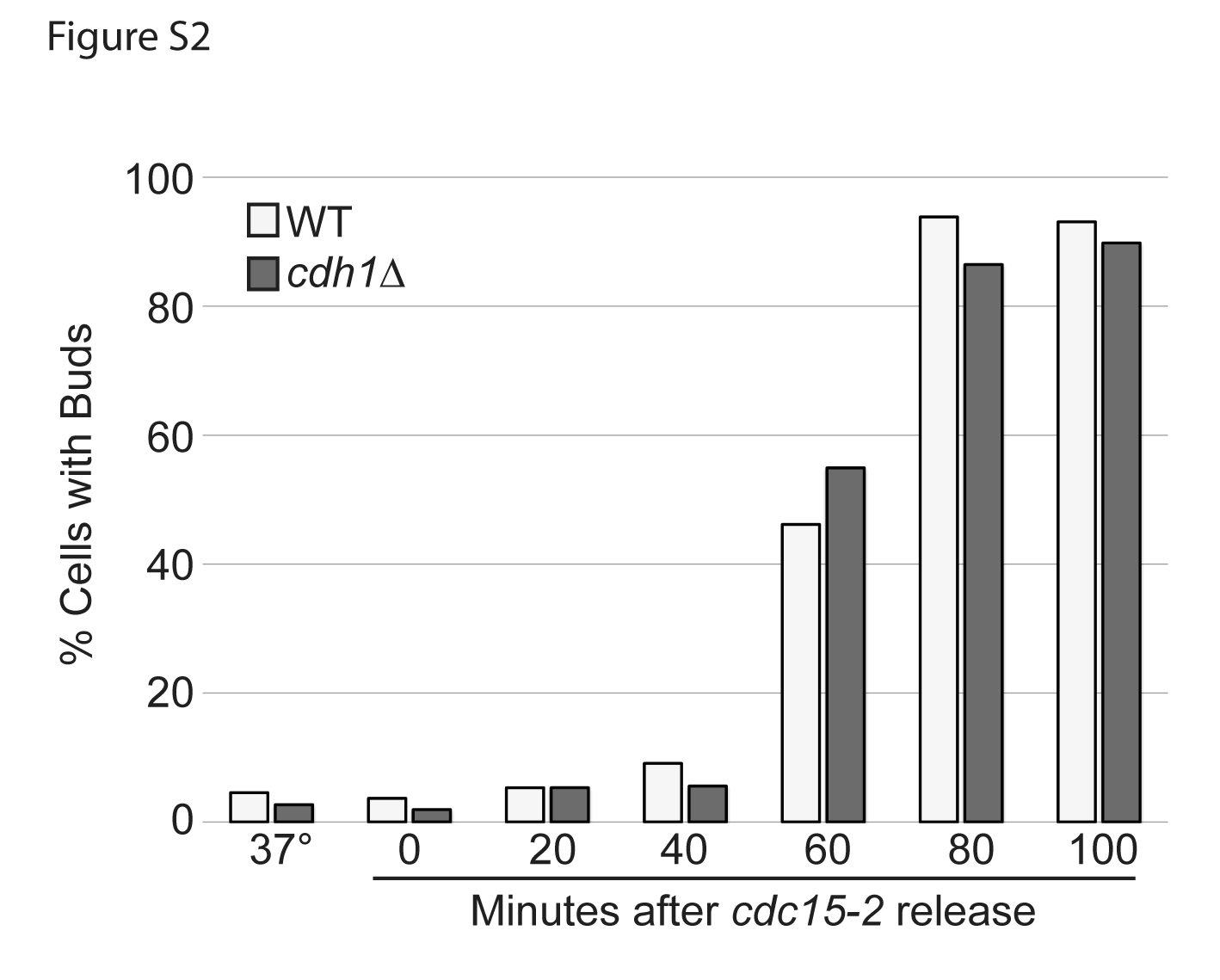

Supplement: Figure S2 — Cells released from the cdc15-2 arrest proceed synchronously into the next cell cycle. Budding index of cells collected at the indicated times after release from the cdc15-2 arrest (Figure 3). Results are from a single WT (light) and cdh1Δ (dark) assay and indicate the percentage of cells with visible buds. This result is representative of the pattern observed from the cdc15-2 arrest and release assays. (TIF) [file pone.0055055.s002.tif]

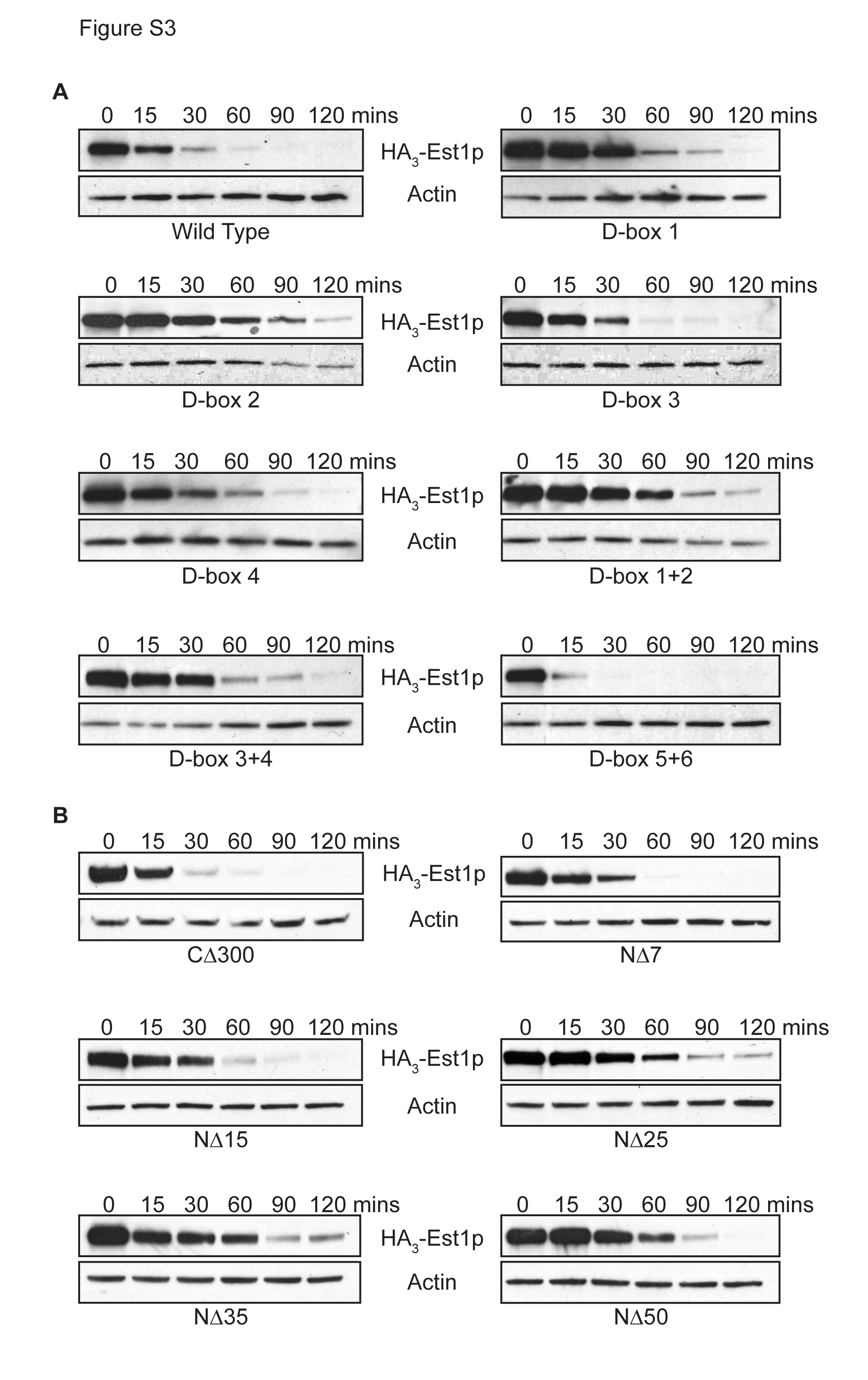

Supplement: Figure S3 — Est1p degradation in G1 phase depends upon specific degron motifs. (A) Western blots of Est1p stability assays from strain YKF802 containing pKF600 (GAL1-HA3-EST1) plasmids expressing the D-box (DB) mutated (RxxL to AxxA) est1 alleles indicated (DB1; DB2; DB1+2; DB3; DB4; DB3+4; DB5+6), treated as in Figure 1B (α-factor). (B) Strain YKF802 containing pKF600 plasmids expressing the deletion variants indicated (CΔ300, NΔ7, NΔ15, NΔ25, NΔ35 or NΔ50) were treated as in (A). Results are quantified in Figure 4. (TIF) [file pone.0055055.s003.tif]

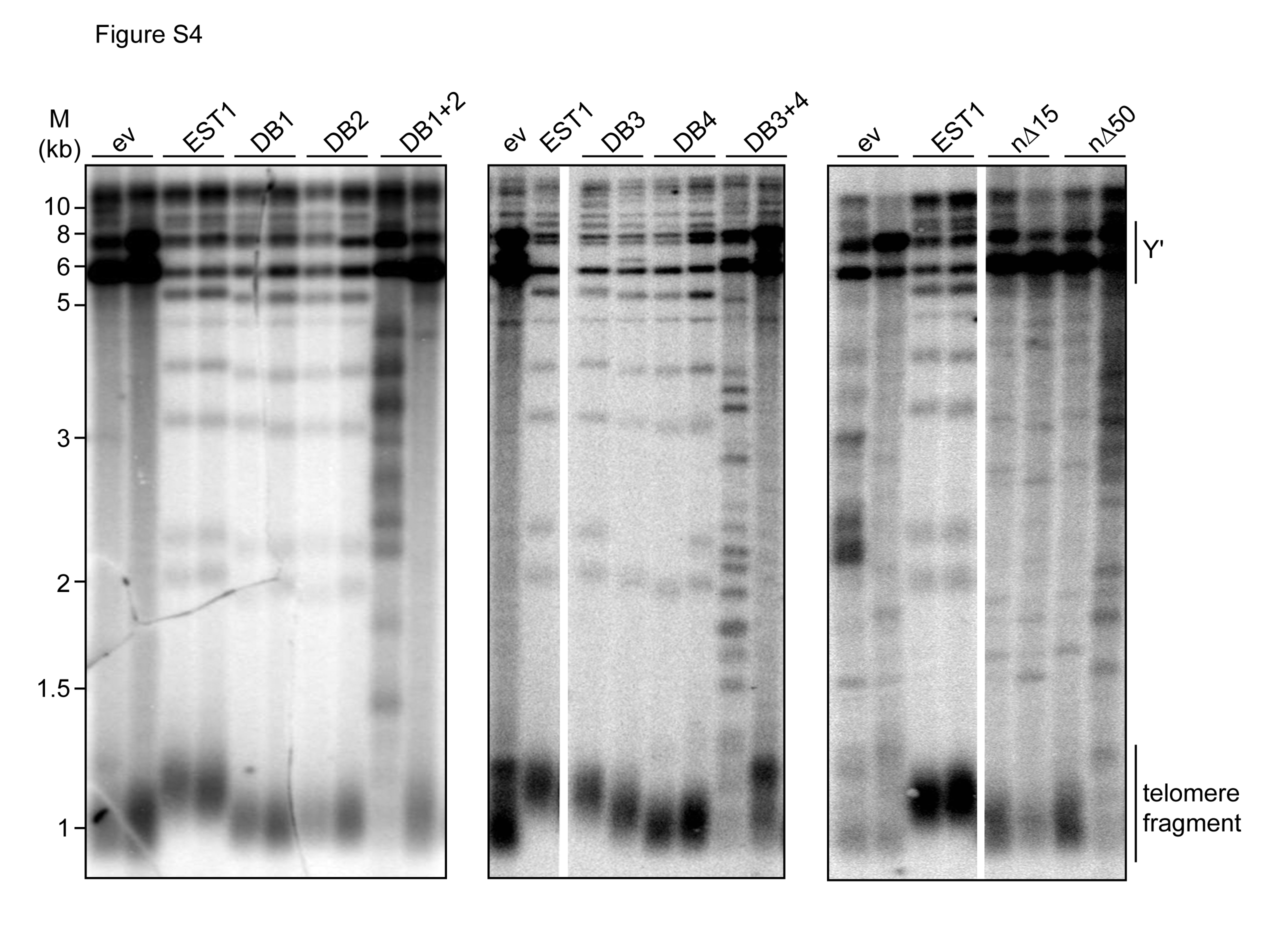

Supplement: Figure S4 — Stabilized alleles of Est1p fail to complement an est1 deletion. Independent isolates from strain YKF810 (est1Δ) harboring plasmids pRS416 (empty vector: ev), pRS416-EST1 (EST1), or the est1 alleles indicated (DB1; DB2; DB1+2; DB3; DB4; DB3+4, NΔ15; NΔ50) were propagated for >100 generations. DNA was extracted, digested with XhoI, Southern blotted, and probed with a randomly labeled telomeric DNA probe. Y′-elements and telomere fragments from Y′-containing chromosomes are indicated. Positions of molecular weight markers (M) are indicated in kilobases (kb). Alleles partially compromised for function have telomere fragments that are shorter than the wild-type control while severely compromised alleles result in the formation of telomerase-negative survivors characterized by Y′-element amplification and/or heterogeneous telomere length (smears throughout the lane). (TIF) [file pone.0055055.s004.tif]
