# Supplementary material for: The Anaphase Promoting Complex Contributes to the Degradation of the S. cerevisiae Telomerase Recruitment Subunit Est1p
Source: PLoS One. 2013 Jan 25;8(1):e55055. doi: 10.1371/journal.pone.0055055 (PMC3555863; doi:10.1371/journal.pone.0055055)
Supplement: Table S1 — S. cerevisiae strains used in this study. (DOCX) [file pone.0055055.s005.docx]

**Table S1:** *S. cerevisiae* strains used in this study.

| **Name** | **Relevant Genotype^a^** | **Source** |
| --- | --- | --- |
| K1534 | *bar1Δ::hisG* | [[1](#_ENREF_1)] |
| YKF800 | *bar1Δ::kanMX4* | This study |
| YKF801 | YKF800 *EST1-MYC_13_* [*hphNT1*] | This study |
| YKF802 | *bar1Δ::hphMX4* | This study |
| K4438 | K1534 *cdc16-123* | [[1](#_ENREF_1)] |
| YKF803 | YKF802 *apc9Δ::kanMX4* | This study |
| YKF804 | YKF802 *mnd2Δ::kanMX4* | This study |
| YKF805 | YKF802 *swm1Δ::kanMX4* | This study |
| MAY6810 | *bar1Δ::hisG clb2Δ::URA3* | [[2](#_ENREF_2)] |
| YKF806 | *bar1Δ:: hphMX4 clb2Δ::URA3* | This study |
| MAY6812 | *bar1Δ::hisG clb2Δ::URA3 cdh1Δ:: KAN^R^* | [[2](#_ENREF_2)] |
| YKF807 | *bar1Δ::hphMX4 clb2Δ::URA3 cdh1Δ::KAN^R^* | This study |
| YCM191 | *bar1Δ::URA3 cdc15-2* | Gift from C. Hardy |
| YKF808 | YCM191 *EST1-MYC_13_ [hphNT1]* | This study |
| YKF809 | YKF808 *cdh1Δ::KAN^R^* | This study |
| YKF810 | YKF802 *est1Δ::kanMX6* | This study |

^a^ All strains are derivatives of W303: *MATa ade2-1 trp1-1 can1-100 leu2-3,112 his3-11,15 ura3 ssd1 GAL*

References

1. Irniger S, Nasmyth K (1997) The anaphase-promoting complex is required in G1 arrested yeast cells to inhibit B-type cyclin accumulation and to prevent uncontrolled entry into S-phase. J Cell Sci 110 ( Pt 13): 1523-1531.

2. Hildebrandt ER, Hoyt MA (2001) Cell cycle-dependent degradation of the *Saccharomyces cerevisiae* spindle motor Cin8p requires APC(Cdh1) and a bipartite destruction sequence. Mol Biol Cell 12: 3402-3416.
