# Supplementary material for: The Anaphase Promoting Complex Contributes to the Degradation of the S. cerevisiae Telomerase Recruitment Subunit Est1p
Source: PLoS One. 2013 Jan 25;8(1):e55055. doi: 10.1371/journal.pone.0055055 (PMC3555863; doi:10.1371/journal.pone.0055055)
Supplement: Table S2 — Plasmids used in this study. (DOCX) [file pone.0055055.s006.docx]

**Table S2:** Plasmids used in this study.

| **Name** | **Relevant Sequence or description** | **Source** |
| --- | --- | --- |
| pVL242RtoA | *P_GAL1_-HA_3_-EST1 [LEU2 2μ]* | [[1](#_ENREF_1)] |
| pKF600 | *P_GAL1_-HA_3_-EST1 [LEU2 2μ]* | This study |
| pKF600-DB1 | *P_GAL1_-HA_3_-est1^D-box 1 (RAHL 🡪 AAHA)^* | This study |
| pKF600-DB2 | *P_GAL1_-HA_3_-est1^D-box 2 (RCFL 🡪 ACFA)^* | This study |
| pKF600-DB3 | *P_GAL1_-HA_3_-est1^D-box 3 (RGAL 🡪 AGAA)^* | This study |
| pKF600-DB4 | *P_GAL1_-HA_3_-est1^D-box 4 (RRRL 🡪 ARRA)^* | This study |
| pKF600-DB1+2 | *P_GAL1_-HA_3_-est1^D-box 1+2^* | This study |
| pKF600-DB3+4 | *P_GAL1_-HA_3_-est1^D-box 3+4^* | This study |
| pKF600-DB5+6 | *P_GAL1_-HA_3_-est1^D-box 5+6 (RSIL 🡪 ASIA and RSYL 🡪 ASYA)^* | This study |
| pKF600-C300 | *P_GAL1_-HA_3_-est1^CΔ300^* | This study |
| pKF600-N7 | *P_GAL1_-HA_3_-est1^NΔ7^* | This study |
| pKF600-N15 | *P_GAL1_-HA_3_-est1^NΔ15^* | This study |
| pKF600-N25 | *P_GAL1_-HA_3_-est1^NΔ25^* | This study |
| pKF600-N35 | *P_GAL1_-HA_3_-est1^NΔ35^* | This study |
| pKF600-N50 | *P_GAL1_-HA_3_-est1^NΔ50^* | This study |
| pRS416 | Empty vector *[URA3 CEN]* | [[2](#_ENREF_2)] |
| pRS416-EST1 | *P_EST1_-EST1* | This study |
| pRS416-DB1 | *P_EST1_- est1^D-box 1 (RAHL 🡪 AAHA)^* | This study |
| pRS416-DB2 | *P_EST1_- est1^D-box 2 (RCFL 🡪 ACFA)^* | This study |
| pRS416-DB3 | *P_EST1_- est1^D-box 3 (RGAL 🡪 AGAA)^* | This study |
| pRS416-DB4 | *P_EST1_- est1^D-box 4 (RRRL 🡪 ARRA)^* | This study |
| pRS416-DB1+2 | *P_EST1_- est1^D-box 1+2^* | This study |
| pRS416-DB3+4 | *P_EST1_- est1^D-box 3+4^* | This study |
| pRS416-N15 | *P_EST1_- est1^NΔ15^* | This study |
| pRS416-N50 | *P_EST1_- est1^NΔ50^* | This study |
| pRS416-CDC16 | *P_CDC16_-CDC16* | This study |
| pKF601 | *P_Sp6_-EST1* | This study |
| pCS2FA2R-Cyclin B | *P_Sp6_-CycB* | Gift from L. Lee |
| pKF602 | *P_T7_-EST1* | This study |
| pRSET-PDS1 | *P_T7_-PDS1* | [[3](#_ENREF_3)] |

References

1. Osterhage JL, Talley JM, Friedman KL (2006) Proteasome-dependent degradation of Est1p regulates the cell cycle-restricted assembly of telomerase in *Saccharomyces cerevisiae*. Nat Struct Mol Biol 13: 720-728.

2. Sikorski RS, Hieter P (1989) A system of shuttle vectors and yeast host strains designed for efficient manipulation of DNA in *Saccharomyces cerevisiae*. Genetics 122: 19-27.

3. Passmore LA, McCormack EA, Au SWN, Paul A, Willison KR, et al. (2003) Doc1 mediates the activity of the anaphase-promoting complex by contributing to substrate recognition. Embo J 22: 786-796.
